# 新型CT定位法在肺癌患者颈内静脉入路输液港植入术中的应用效果

**DOI:** 10.3779/j.issn.1009-3419.2026.102.23

**Published:** 2026-08-20

**Authors:** Dian REN, Wenhao ZHAO, Dinghuan ZHAO, Tong LI, Yu HUA, Jing WANG, Jiachen HU, Yajun WANG, Song DU, Lingyu GUO, Yajie CUI, Mei LI, Huihui JI, Yi SHI, Xin LI, Jun CHEN

**Affiliations:** 1 300052 天津，天津医科大学总医院肺部肿瘤外科; 1 Department of Lung Cancer Surgery, Tianjin Medical University General Hospital, Tianjin 300052, China; 2 300052 天津，天津医科大学总医院，天津市肺癌研究所，天津市肺癌转移与肿瘤微环境重点实验室; 2 Tianjin Key Laboratory of Lung Cancer Metastasis and Tumor Microenvironment, Tianjin Lung Cancer Institute, Tianjin Medical University GeneralHospital, Tianjin 300052, China; 3 300070 天津，天津医科大学; 3 Tianjin Medical University, Tianjin 300070, China; 4 300022 天津，天津市眼科医院麻醉科; 4 Department of Anesthesiology,Tianjin Eye Hospital, Tianjin 300022, China

**Keywords:** 肺肿瘤, 输液港, 导管长度, CT定位, 尖端定位, 中心静脉, Lung neoplasms, Totally implantable venous access port, Catheter length, CT-based localization, Catheter tip positioning, Central venous access

## Abstract

**背景与目的:**

输液港可显著降低肺癌化疗人群中静脉通路相关并发症的发生率，但其导管尖端精准置入上腔静脉-右心房交界处（cavoatrial junction, CAJ）仍是手术关键技术难点。目前，常用的心电图（electrocardiogram, ECG）引导定位法操作复杂、耗时较长。本研究基于临床实践，提出一种新型计算机断层扫描（computed tomography, CT）定位法，并科学评估该新型定位法的临床效能。

**方法:**

回顾性分析2024年1月至2026年4月于天津医科大学总医院肺部肿瘤外科行颈内静脉入路输液港植入的40例肺癌患者，根据定位方法分为CT定位组（*n*=20）与ECG定位组（*n*=20）。通过术后CT测量导管尖端和CAJ距离，比较两组术后导管尖端位置的准确性和一致性，同时统计穿刺点和CAJ对应的体表解剖标志。

**结果:**

CT定位组定位精度更高、结果离散度更低（*P*=0.009）；颈内静脉穿刺点对应的颈椎棘突水平以第7颈椎（C7）棘突最为常见，CAJ的体表投影在前胸壁和背部分别集中于第4肋-第4肋间隙和第7-8肋间隙。

**结论:**

新型CT定位法可显著提高颈内静脉内置管的准确率，优于传统ECG定位法，值得临床推广。

## Abstract (en)

**Background and objective:**

Implantable venous ports can substantially reduce venous access-related complications in patients undergoing chemotherapy for lung cancer. However, accurate positioning of the catheter tip at the cavoatrial junction (CAJ), defined as the anatomical junction between the superior vena cava (SVC) and right atrium, remains technically challenging. Although electrocardiography (ECG)-guided positioning is widely used in clinical practice, the technique can be relatively complex and time-consuming. Based on clinical practice, we developed a novel computed tomography (CT)-based positioning technique and aimed to evaluate its clinical efficacy and positioning accuracy.

**Methods:**

We retrospectively analyzed 40 patients with lung cancer who underwent implantable port placement via the internal jugular vein at the Department of Lung Cancer Surgery, Tianjin Medical University General Hospital, between January 2024 and April 2026. The patients were divided into two groups according to the catheter tip positioning technique: the CT-guided positioning group (*n*=20) and the ECG-guided positioning group (*n*=20). Postoperative CT imaging was performed to measure the distance between the catheter tip and the CAJ. The accuracy and variability of catheter tip positioning were compared between the two groups. In addition, the surface anatomical landmarks corresponding to the internal jugular vein puncture site and the CAJ were recorded and analyzed.

**Results:**

Compared with the ECG-guided group, the CT-guided group demonstrated significantly greater catheter tip positioning accuracy and lower positional variability (*P*=0.009). The seventh cervical spinous process (C7) was the most common surface anatomical landmark corresponding to the internal jugular vein puncture site. The surface projection of the CAJ was predominantly located at the level of the 4^th^ costal cartilage and the 4^th^ intercostal space on the anterior chest wall and the 7^th^-8^th^ costal cartilage level on the posterior thoracic wall.

**Conclusion:**

The novel CT-based positioning technique demonstrated greater accuracy and consistency in catheter tip placement than ECG-guided positioning in patients undergoing internal jugular vein port implantation.

肺癌是中国发病率和死亡率均居首位的恶性肿瘤，2011年全国肿瘤登记年报数据^[1]^显示，肺癌新发病例与死亡病例数均位居所有恶性肿瘤之首，且在男性及城市地区人群中尤为突出。中晚期肺癌患者主要接受以化疗为主的综合治疗。然而，长期静脉化疗易导致化疗药物外渗、静脉炎、血管内膜损伤及继发性血管硬化等并发症，不仅增加导管相关不良事件风险，亦可能影响治疗连续性与患者生活质量。静脉输液港是保障中晚期肺癌患者长期、安全静脉给药的关键通路装置。其中，颈内静脉入路因解剖路径直、穿刺成功率高、血栓形成及感染发生率较低，且患者舒适度与依从性更优，已成为当前临床实践中输液港植入的首选路径^[2,3]^。

手术成功的关键在于导管尖端精确定位于上腔静脉下1/3段至上腔静脉-右心房交界处（cavoatrial junction, CAJ），该位置可保障药物迅速稀释，降低血管内皮损伤风险^[4]^。目前，介入放射科X线透视引导定位被公认为导管尖端定位的金标准，但受限于设备依赖性强、操作环境要求高及电离辐射暴露风险，临床普及率较低。术中心电图（electrocardiogram, ECG）P波振幅变化法因操作便捷、实时性强，已成为主流辅助定位技术^[5]^。然而，ECG定位法存在固有局限：个体解剖变异（如上腔静脉长度、心包反折位置）会导致导管预置长度差异大；同时，ECG定位需术中同步监测和判读，须两人协作，无法单人操作，增加人力成本并延长手术时间。基于长期临床实践，本研究团队提出一种融合术前体表测量与胸部计算机断层扫描（computed tomography, CT）影像解剖标志定位的新方法，通过术前CT图像识别CAJ的相对体表解剖标志作为测量起点，将术前穿刺点作为测量终点，建立个体化导管预置长度预测模型，以提高颈内静脉内置管的准确率。

## 1 资料与方法

### 1.1 一般资料

本研究为回顾性队列研究，研究对象为确诊肺鳞癌需化疗行颈内静脉入路输液港植入的患者。鉴于天津医科大学总医院肺部肿瘤外科自2025年1月起全面采用CT引导下导管尖端定位技术，替代既往常规使用的ECG定位法，本研究据此划分两组：2024年1月至2025年1月接受ECG定位法完成输液港导管尖端定位的肺癌患者归为ECG定位组（*n*=20）；2025年1月至2026年4月接受CT定位法完成尖端定位的肺癌患者归为CT定位组（*n*=20）。纳入标准：（1）首次接受输液港植入术；（2）年龄≥18周岁；（3）无严重凝血功能障碍[国际标准化比值（international normalized ratio, INR）<2.0且血小板计数>50×10^9^/L]；（4）术后完成胸部平扫CT复查；（5）签署知情同意书。排除标准：（1）既往同侧颈内静脉置管史；（2）活动性静脉血栓形成或确诊上腔静脉综合征；（3）严重脊柱畸形；（4）临床及影像学资料不完整，无法准确判读导管尖端位置者。本研究已获天津医科大学总医院伦理委员会批准（批准号：IRB2024-YX-045-01）。

### 1.2 定位与手术方法

（1）CT定位组：术前获取患者平卧位胸部CT（层厚≤1.25 mm），在横断面图像上精确定位CAJ；同步识别CAJ所在层面的肋骨-胸骨交界点（即胸骨角对应肋软骨连接处），作为体表解剖标志点。术中患者取仰卧位，采用高频线阵超声探查颈内静脉走行及穿刺路径，确定最优穿刺点；以直尺沿矢状轴平行于脊柱长轴及水平面，测量穿刺点至体表肋骨-胸骨交界点的直线距离；该距离减去7 mm即为预设导管置入长度（图1）。（2）ECG定位组：术中实时监测标准II导联ECG，当导管推进过程中P波振幅达R波振幅50%时，判定导管尖端已达CAJ水平，此时停止推进，记录并确认导管置入长度。

**图1:**
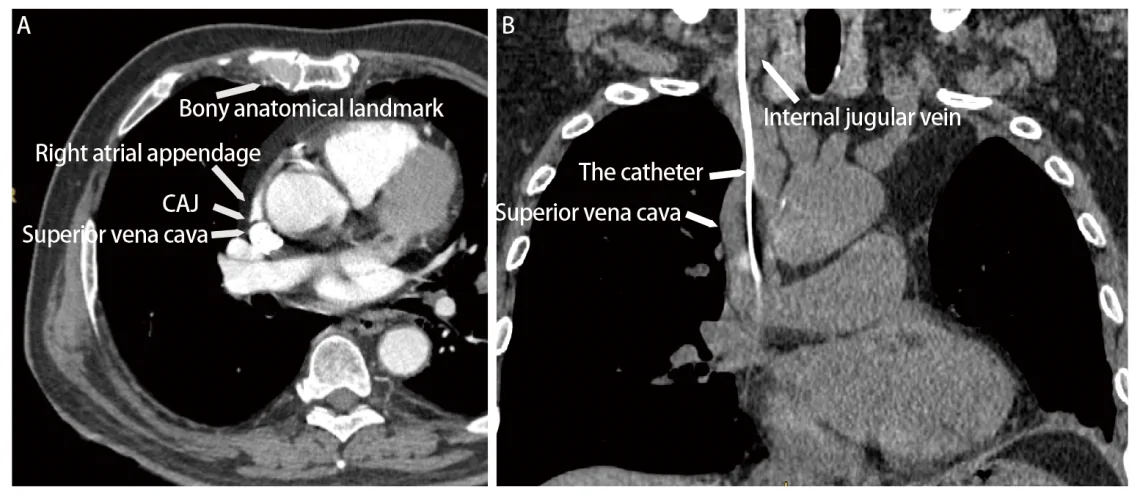
CT定位法原理展示。A：CT定位法中CAJ和骨性解剖标记的定位示意图；B：当颈内静脉与上腔静脉呈S形走行时，导管仍沿人体长轴呈直线走行。

### 1.3 观察指标

主要结局指标：基于术后复查的胸部平扫CT图像，由两名独立放射科医师（均具备5年以上胸部影像诊断经验，且对分组信息设盲）采用双盲法测量导管尖端至CAJ的垂直距离，取二者均值作为最终结果；若差异>2 mm，则由第三位高级职称医师裁定。次要结局指标：（1）实际导管置入长度；（2）导管上缘及尖端在体表的投影位置；（3）导管尖端是否误入右心房（定义为尖端超过CAJ平面进入右心房腔内>5 mm）；（4）术中出血量；（5）术后30天内感染（包括穿刺点感染、隧道感染及其他导管相关感染）等围术期并发症的发生率。

### 1.4 统计学方法

采用SPSS 21.0软件进行统计分析。连续变量首先进行*Shapiro-Wilk*正态性检验；符合正态分布者以均数±标准差（Mean±SD）表示，组间比较采用独立样本*t*检验；不符合正态分布者以中位数[M (Q_1_, Q_3_)]表示，组间比较采用*Mann-Whitney U*检验。分类变量以频数（百分比）[*n*（%）]表示，组间比较采用*Pearson *χ²检验；当任一格子理论频数<5时，改用*Fisher*确切概率法。所有检验均为双侧检验，以*P*<0.05为差异具有统计学意义。

## 2 结果

### 2.1 两组基线资料比较

CT定位组与ECG定位组在性别、年龄、身高、身体质量指数（body mass index, BMI）及病理分期等基线指标上差异均无统计学意义（*P*>0.05）。其中，两组性别构成比无统计学差异（*P*>0.05），但整体样本中男性占比90.0%（36/40），主要源于本中心收治人群均为需紫杉醇类化疗的肺鳞癌患者，该病理类型男性发病率显著高于女性（表1）。

**表1:** 两组患者基线数据的比较

Variable	CT-guided positioning group (*n*=20)	ECG-guided positioning group (*n*=20)	*t*/*Z*/*χ²*	*P*
Sex			*χ²*=0.278	0.605
Male	17 (85.00%)	19 (95.00%)		
Female	3 (15.00%)	1 (5.00%)		
Age (yr)	65.50±4.35	68.55±7.34	*t*=-1.598	0.120
Height (m)	1.70 (1.65, 1.74)	1.70 (1.68, 1.74)	*Z*=178.500	0.566
BMI (kg/m^2^)	23.55±2.12	24.45±2.99	*t*=-1.088	0.284
Pathological stage			*χ²*=1.829	0.665
Stage IB	5 (25.00%)	3 (15.00%)		
Stage II	1 (5.00%)	1 (5.00%)		
Stage III	12 (60.00%)	11 (55.00%)		
Stage IV	2 (10.00%)	5 (25.00%)		

ECG: electrocardiogram; BMI: body mass index.

### 2.2 两组患者结局指标对比

CT定位组导管尖端至CAJ的距离显著小于ECG定位组，差异具有统计学意义（*P*=0.009）。CT定位组的四分位间距（9.38 mm）明显窄于ECG定位组（18.75 mm），提示其定位精度更高，结果离散度更低。颈内静脉穿刺点对应的颈椎棘突水平以第7颈椎（C7）棘突最为常见，占全部40例患者的77.50%；CAJ的体表投影在前胸壁主要集中于第4肋-第4肋间隙，占比80.0%；在背部则主要投影于第7-8肋，合计占比90.0%（表2）。

**表2:** 两组患者结局指标的比较

Variable	CT-guided positioning group (*n*=20)	ECG-guided positioning group (*n*=20)	*t*/*Z*/*χ*²	*P*
Tip to CAJ distance (mm)	3.75 (0.00, 9.38)	14.38 (4.38, 23.13)	*Z*=104.000	0.009
Surface positioning of the upper edge of the catheter			*χ²*=1.532	0.695
C7	15 (75.00%)	16 (80.00%)		
C6	5 (25.00%)	3 (15.00%)		
T1	0 (0.00%)	1 (5.00%)		
CAJ surface projection site on the anterior chest wall			*χ²*=3.365	0.394
The 3^rd^ intercostal space	3 (15.00%)	3 (15.00%)		
The 4^th^ rib	10 (50.00%)	8 (40.00%)		
The 4^th^ intercostal space	5 (25.00%)	9 (45.00%)		
The 5^th^ rib	2 (10.00%)	0 (0.00%)		
CAJ surface projection site on the posterior chest wall			*χ²*=4.895	0.354
The 6^th^ intercostal space	3 (15.00%)	0 (0.00%)		
The 7^th^ rib	6 (30.00%)	6 (30.00%)		
The 7^th^ intercostal space	7 (35.00%)	6 (30.00%)		
The 8^th^ rib	4 (20.00%)	7 (35.00%)		
The 8^th^ intercostal space	0 (0.00%)	1 (5.00%)		
Catheter insertion length (cm)	14.85±0.76	14.20±1.23	*t*=2.010	0.053

### 2.3 手术相关并发症发生情况

两组患者均未发生导管尖端误入右心房、术中明显出血（>50 mL）、术后30天内穿刺点感染、隧道感染或导管相关血流感染等手术操作相关并发症。

## 3 讨论

输液港适配化疗、靶向/免疫治疗等多周期给药需求，可耐受高浓度化疗药物输注，尤其在以紫杉醇类药物为主的方案中，显著降低血管损伤与药物外渗风险。其全植入式设计使其导管相关感染、深静脉血栓及其他不良事件风险大大降低，患者生活质量更高^[6,7]^。因并发症发生率低、维护需求少、使用寿命长，长期应用具有明确的成本效益优势^[8]^。临床需结合治疗周期（≥3个月优先）、解剖条件（如颈部放疗史选对侧入路）个体化选择，是肺癌患者安全高效的静脉通路优选。

输液港导管尖端精确定位是核心环节，上腔静脉由左、右头臂静脉汇合而成，CAJ被公认为导管尖端的理想置入靶点^[9]^。导管长度偏差是导致并发症的关键可控因素：长度过短易致药物流速过快、局部血流动力学紊乱，显著增加血管内皮损伤及血栓形成风险；研究^[10]^显示，尖端位于上腔静脉以外者血栓发生率达15%，显著高于位置正确者（5%）；长度过长则可能引发心肌刺激、室性早搏甚至房室传导阻滞，发生率为1%-3%^[11]^；此外，长度不当亦与导管功能障碍（如堵塞、移位、断裂）密切相关。

当前临床实践中，体表测量法联合术后X线平片验证仍为常用策略，但其受个体解剖变异影响较大。一项回顾性研究^[12]^提出以胸部X线测量“C长度”（右第1胸椎横突至气管隆嵴距离）指导右锁骨下静脉入路输液港置管，122例患者中导管尖端全部成功定位至上腔静脉内；另一项前瞻性研究^[11]^通过经食管超声心动图测定52例心脏手术患者右颈内静脉至CAJ的距离，均值为（17.8±1.3）cm（范围：15.0-20.0），且该距离与身高呈弱相关（*r*=0.28）。另有研究^[13]^提出，在成人患者中，可将胸骨长度的中点作为体表解剖标志，辅助判断右侧CAJ的大致水平位置。近年虽有研究尝试将CT用于术前规划，但多数未证实其相较传统方法具有显著优势。基于上述背景与实证分析，本研究提出一种新型术前CT引导定位策略，依托高分辨率胸部CT（层厚≤1.25 mm），精准识别CAJ三维空间坐标及穿刺点体表投影，计算矢状面投影距离并校正7 mm，实现导管长度的个体化预设。

本研究结果提示，CAJ体表投影在前胸壁主要集中于第3-5肋间隙，以第4肋-第4肋间隙最为常见（80.0%, 32/40）。为了避免导管误入心房，建议将导管放置于第3肋间隙，该结论和既往研究^[14]^一致。然而，既往研究^[14]^提示：当导管尖端体表投影对第3肋间隙时，其实际位于CAJ附近的概率为95.0%，本研究结果提示将导管放置于第3肋间隙时，可以基本满足将导管尖端放置于上腔静脉中下段而非CAJ。本研究认为该差异主要源于CAJ测量方法的不同：既往研究依赖X线平片结合心影轮廓进行粗略估算，而CT则可以将CAJ的位置精确到毫米级别，故所确定的CAJ平面更靠近心脏基底部。本研究首次系统验证并明确了CAJ对应的后胸壁体表标志——以第7-8肋为主要参照，拓展了传统前胸壁定位体系，为临床提供多维度体表定位依据。

上腔静脉内ECG定位法在癌症患者中心导管定位中有着广泛的应用^[15,16]^，定位过程中心电图表现有四种类型：P波增高、P波阴性加深、P波无变化，以及心房颤动/心房扑动。其中P波增高最常见^[16]^，出现其他三种情况时，则可能会造成定位失败。Lv等^[17]^的研究通过CT三维重建的方法分段预测导管长度，相比ECG定位法有更好的精准度。但是术前需要拷贝CT原始图像，由专人进行三维重建，分段测量后再进行相加，操作过于繁杂，临床应用受到限制。本研究的测量方法无需进行复杂的重建及计算过程，更适合临床推广，另外导管在血管中并不沿血管中心线走行，不认可Lv等^[17]^将中心线的测量距离作为导管的预测长度。

综上所述，相较于传统ECG定位法，新型CT引导术前精准规划定位法可显著提高颈内静脉入路输液港导管尖端抵达CAJ靶区的成功率与一致性，且不增加围术期并发症风险。该方法将影像诊断优势转化为外科操作精度，具备良好的临床实用性与推广价值，值得在具备CT条件的肿瘤中心，特别是肺癌专科诊疗单位中规范应用。

本研究存在若干局限性：首先，回顾性队列设计可能存在选择偏倚；其次，受限于纳入标准，样本量相对有限。此外，CT检查涉及辐射暴露与额外费用，需权衡获益风险比；但需指出，对于肺癌患者，胸部CT为疾病分期与疗效评估的必查项目；对其他实体瘤患者，胸部CT亦是远处转移筛查的常规手段。因此，该技术适用于需规律影像随访的肿瘤患者，尤其推荐用于肺癌患者的输液港植入。
